# A Renaissance for Oncolytic Adenoviruses?

**DOI:** 10.3390/v15020358

**Published:** 2023-01-26

**Authors:** Paola Blanchette, Jose G. Teodoro

**Affiliations:** 1 Goodman Cancer Institute, McGill University, Montréal, QC H3A 1A3, Canada; 2 Department of Biochemistry, McGill University, Montréal, QC H3G 1Y6, Canada; 3 Department of Microbiology and Immunology, McGill University, Montréal, QC H3G 1Y6, Canada

**Keywords:** Adenovirus, oncolytic viruses, immune checkpoint inhibitors, clinical trials, cancer therapy

## Abstract

In the 1990s, adenovirus became one of the first virus types to be genetically engineered to selectively destroy cancer cells. In the intervening years, the field of “oncolytic viruses” has slowly progressed and culminated in 2015 with the FDA approval of Talimogene laherparepvec, a genetically engineered herpesvirus, for the treatment of metastatic melanoma. Despite the slower progress in translating oncolytic adenovirus to the clinic, interest in the virus remains strong. Among all the clinical trials currently using viral oncolytic agents, the largest proportion of these are using recombinant adenovirus. Many trials are currently underway to use oncolytic virus in combination with immune checkpoint inhibitors (ICIs), and early results using oncolytic adenovirus in this manner are starting to show promise. Many of the existing strategies to engineer adenoviruses were designed to enhance selective tumor cell replication without much regard to interactions with the immune system. Adenovirus possesses a wide range of viral factors to attenuate both innate anti-viral pathways and immune cell killing. In this review, we summarize the strategies of oncolytic adenoviruses currently in clinical trials, and speculate how the mutational backgrounds of these viruses may impact upon the efficacy of these agents in oncolytic and immunotherapy. Despite decades of research on human adenoviruses, the interactions that these viruses have with the immune system remains one of the most understudied aspects of the virus and needs to be improved to rationally design the next generation of engineered viruses.

The first example of a virus that was genetically modified to selectively replicate in cancer cells came from a 1991 study of Herpes Simplex Virus 1(HSV-1) [1]. In the case of HSV-1 the genetic modification of virus was a deletion of the thymidine kinase gene, which made the virus dependent upon infecting dividing cells that maintain higher nucleotide pools. The development of HSV-1 as an oncolytic moved through several iterations, culminating in the development and FDA approval of Talimogene laherparepvec (TVEC) in 2015 for the treatment of melanoma. In addition to deleting viral genes involved in virus pathology and immune suppression, TVEC also included the strategy of arming the virus with a cellular gene, GM-CSF, to stimulate immunity and mount an immune response against the tumor. Whereas early research to optimize oncolytic viruses focussed on enhancing differential expression between normal and tumor cells, the current emphasis has shifted towards maximizing immune responses against tumor cells infected with the oncolytic virus. The clinical success of immune checkpoint inhibitors (ICIs) has diverted some attention away from the use of viral agents as monotherapy agents; however, the limitations of ICIs have also raised opportunities for combination therapy with oncolytic viruses. Cancer therapies with ICIs typically have a major portion of patients that fail to respond because the tumors do not have sufficient immune infiltration and are immunologically “cold”. The use of viral agents to turn immunologically “cold” tumors “hot” and improve ICI therapeutic response has raised interest in the use of oncolytic viruses [2]. The ability of recombinant viruses to be armed with transgenes expressing pro-inflammatory proteins makes them ideal tools to couple with ICI therapy. The clincicaltrials.gov website currently shows that there are 130 ongoing clinical trials using oncolytic viruses, and of these 33 are testing combinations with a viral oncolytic and ICIs.

Although TVEC was the first, and currently the only, US FDA approved oncolytic virus, it was not the first to be approved in the world. The first oncolytic virus to be approved was H101 (oncorin), an adenoviral oncolytic, which was approved in 2005 by the Chinese State Food and Drug Administration for the treatment of head and neck cancers [3]. The design strategy for H101 came from previous studies from the lab of Frank McCormick, which showed that a mutant virus carrying a deletion of the E1B-55K gene in Adenovirus type 5 (Ad5), later called Onyx-015, resulted in selective viral replication in cancer cells with p53 mutations [4]. The E1B-55K gene encodes a viral protein that binds and inhibits the p53 protein to prevent virus induced apoptosis and cell cycle arrest. Since the publication of this study, the mechanism of action of the Onyx-015 virus has been disputed, and alternative mechanisms such as differences in mRNA transport have been proposed [5]. Although the clinical application of Onyx-015 was not pursued further, H101 has multiple clinical trials underway targeting a variety of cancer types. Despite the challenges posed by using adenovirus as an oncolytic agent, interest in the virus for clinical applications remains strong. Examining the 130 oncolytic virus clinical trials listed on the clincicaltrials.gov website shows that the largest fraction of these (31%) utilize Adenovirus, followed by HSV (28%) and Vaccinia (21%) (Figure 1).

There are several properties of human adenoviruses that make them ideally suited as next generation oncolytic agents used in combination with ICIs. First, the molecular virology of adenovirus has been studied for several decades, and it is one of the most well characterized viral families. Second, deriving recombinant adenovirus has become relatively straightforward. Importantly, most individuals are seropositive for Adenovirus type 5 (Ad5), the most commonly used serotype as an oncolytic agent [6]. The use of Ad5 based vectors for vaccine delivery or as gene vectors is complicated by the high seropositivity in the population; however, this is likely a beneficial property when considering Adenovirus in combination with ICIs. Since most cancer patients will be seropositive for Ad5, they will be able to rapidly mount a response against infected tumor cells. Early data using oncolytic Ad5 in combination with ICIs are beginning to show promise. At the recent American Association for Cancer Research (AACR) and ASCO 2022 meetings, the biotech company GC Oncology presented striking clinical phase I/II data using an oncolytic Ad5 (CG0070) in combination with the ICI, Keytruda (Pembrolizumab). Although the cohort consisted of only 16, patients, 14 of these achieved a complete response after only three months of treatment [7]. The development of adenovirus-based vectors as oncolytic agents has progressed through several generations and 20 different genetically engineered version of vectors have been recently used in clinical trials (Table 1). These recombinant adenoviruses are in use in 42 clinical trials (Table 2). Several modifications have been made to the viral genome in efforts to make the replication of the virus more selective to cancer cells. In some cases, oncolytic adenovirus (OAd) vectors have been armed with cellular genes to enhance immune responses. Two OAds include the cDNA encoding Granulocyte-Macrophage Colony-Stimulating Factor (GM-CSF), a similar strategy utilized in the development of TVEC. Interestingly, CG0070, which has shown promising results in the clinic, also includes a CM-CSF transgene. Other immune stimulating transgenes used include CD40 ligand, TNFα, IFNβ, OXO40 Ligand, IL-2, CXCL9, and CXCL10 (Table 1). 

## 1. Combining Oncolytic Adenovirus with Immune Checkpoint Inhibitors

The rationale for the clinical use of OAd in combination with ICIs is supported by several preclinical studies demonstrating enhanced therapeutic effects. Using an immunohumanized glioblastoma model, an Ad vector encoding a PD-L1 antibody (XVir-N-31) was shown to induce strong immunogenic anti-tumor effects that exceed those with viral treatment alone [31]. Another study using an E1A delta24 OAd expressing a modified version of PD-1 (Ad-CAB) demonstrated excellent immune cell killing of tumor cell both with small cell lung carcinoma [32]. Combination of H101 with anti-PD-1 therapy was shown to enhance anti-tumor response in murine colorectal cancer [33].

Interestingly, even OAd that have already been armed with immune stimulating cytokines can be enhanced even further in combination with ICIs. TILT-123, for example, is armed with TNFa and IL-2 (Table 1) and has been used in combination with PD-1 inhibitors to treat ovarian and head and neck cancers in mouse [34,35]. This same combination was also shown to effectively prevent growth and metastasis of murine melanoma and colorectal cancer [36]. Impressively, metastasis was also inhibited, and mice were rendered resistant to subsequent challenge with injected tumor cells. An OAd armed with GM-CSF was also shown to be greatly enhanced in combination with anti-cytotoxic T lymphocyte-associated antigen-4 (CTLA-4) or anti-PD1 antibodies for the treatment of triple negative breast cancers [37]. Similarly, an OAd expressing GM-CSF, IL12, and relaxin was enhanced by combination with a PD-1 inhibitor in a hamster pancreatic cancer model [38]. The molecular virology of Adenovirus has been studied for 50 years and is well understood; however, the question of which viral genes should be mutated to enhance the oncolytic properties is still an open question. Like many viruses, Adenoviruses also possess an array of viral factors that target immune responses to infected cells and the innate cellular anti-viral pathways such as interferon (IFN) signaling. If Ad-based oncolytic viruses are to be used in combination with ICIs, the mutations introduced in the viral genome need to be re-evaluated for this purpose. Existing laboratory strains of Ad5 commonly contain mutations in the E3 transcription unit of the virus. E3 mutations were often used in the early days of adenovirus research to facilitate molecular cloning of the genome, and since this transcription unit appeared to be dispensable for efficient viral replication in vitro. Subsequent research has shown that the genes encoded by E3 are a multi-functional immune inhibitory cassette that is essential for viral evasion of immune surveillance. To enhance immune stimulating effects of adenovirus, mutating some the E3 functions would certainly be beneficial. In addition to E3, the E1A protein is also known to mediate suppressive effects on IFN-dependent gene expression [39,40,41]. Similarly, adenovirus expresses non-coding RNAs (VA-RNAs), that also attenuates innate anti-viral responses [42,43,44]. Future iterations of OAd should be optimised for selective replication in cancer cells while enhancing pro-inflammatory effects around the tumor. In the sections below we summarize the genetic modifications that are included in OAd vectors currently in clinical trials and provide an assessment of how these mutations can potentially affect the properties of the virus as a mono therapy or in combination with ICIs. Beyond existing OAd vectors, we speculate on other modifications that may enhance tumor selective replication and/or increase anti-tumor immunity.

## 2. Modifications in the E1A Gene

The E1A transcription unit is the first to be expressed during adenovirus infection. The protein encoded on E1A is a critical transcription factor required to drive early viral gene expression and is absolutely required for viral replication. E1A is also oncogenic, inducing cell cycle progression and expression of S-phase genes in preparation for viral DNA replication. Different strategies have been implemented to render replication of the OAds more selective to tumor cells rather than normal cells. One of these was to make E1A ineffective in non-dividing cells. E1A contains two conserved regions (CR1 and CR2) that are required to bind to the tumor suppressor protein Rb to release its interaction from the cellular transcription factors E2Fs. The E2Fs are required for activation of the adenovirus E2 region coding for the viral replication machinery and to enhance expression of cellular S-phase genes. Fueyo et al. [45] deleted the CR2 region (this mutant is now called the delta 24 mutation (deleting 8 aa)), reasoning that a virus with this mutation would kill target tumor cells while spreading the infection within the tumor but not the non-dividing cells surrounding the tumor. This mutation has been frequently adopted in combination with other alterations. Of the 17 different oncolytic viruses described in this study that were not deleted for the entire E1 region, 9 contain this deletion.

Another strategy is to modify the promoter of the E1A gene to make it selectively active in cancer cells. One such strategy was to include E2F regulatory elements in the E1A promoter, which makes the E1A promoter more selective to cells that are already dividing [46]. Another was to make a deletion of 50 bp in the E1A promoter, which resulted in a marked reduction in the expression of both E1A and E1B in growth arrested cells but not in tumor cells [15]. In other cases, the promoter was replaced with that of the telomerase gene [17] or the survivin gene [29].

As mentioned above, E1A protein is also known to mediate suppressive effects on IFN-dependent gene expression [39,40,41]. Although none of the virus mutants in the clinical trials have use the strategy to mutate E1A in a way for it not to interfere with IFN gene expression, it is nonetheless a strategy that could be taken in future studies. The major mechanism for this effect appears to be that E1A represses the transcription of interferon-stimulated genes (ISG) by decreasing the levels of ISRE- binding factors, an effect dependent on the CR1 region [39,40,41]. However, since this region is critical for activating E2F, such mutations may also come with a cost of attenuated viral replication. There are, however, other mechanisms used by E1A. The N-terminal region of E1A (before the CR1 motif) is involved in the binding to hBre1 complex to block IFN-induced H2B mono-ubiquitination that is required for ISG expression [47,48]. The N-terminal region (as well as the CR3) is also involved in binding a component of the immunoproteasome, MECL1 [49]. It is thought that this interaction could result in reduced antigen presentation by the MHCI system [49]. The C-terminal of E1A has been known for some time to bind three proteins, FOXK, DCAF7 and CtBP. More recently, Zemke and Berk have shown that these interactions with E1A result in the downregulation of a subset of ISGs [50]. It may therefore be of interest to determine if mutations in these regions of E1A improve the immunogenicity of OAd vectors.

## 3. Modification of the E1B Gene

The E1B genes have also been modified in some OAds. This transcription unit encodes two different proteins, E1B-55K and E1B-19K, on overlapping reading frames. The E1B-55K was the gene mutated in the first OAd Onyx-015 (see above) as it is required for the inactivation of the p53 tumor suppressor. Two of the viruses that are undergoing clinical trials, Oncorin and MEM-288, have mutations that eliminate expression of E1B-55K. In addition to inactivation of p53, E1B-55K has other viral functions including viral mRNA transport and shut-off of host cell mRNA translation [51]. Viral mutants of E1B-55K do appear to be selectively oncolytic, although the mechanism of this effect is likely more complex than originally hypothesized [52,53].

The E1B-19K gene encodes a functional homolog of the cellular Bcl-2 protein and functions as a suppressor of apoptosis during viral replication [54]. Viral mutants that do not express E1B-19K are more lytic and have much larger plaque sizes when grown in vitro [55]. The E1B-19K gene has been deleted in one vector currently in clinical trials (AdAPT-001). It appears that in this case the E1B-19K gene was removed to insert the transgene in its place, and it is unclear if it was intended to also improve the oncolytic functions of the vector [16]. The impact of E1B-19K on the efficacy of adenovirus as an oncolytic has never been directly addressed in the literature but should be considered. One study has shown that E1B-19K represses pro-inflammatory signaling during viral replication [56]. The corpses of cells killed by virus lacking expression of E1B-19K elicited stronger pro-inflammatory cytokine production when presented to macrophages. This observation suggests that deletion of E1B-19K would be beneficial in the design of an OAd to enhance immune infiltration of tumors and may also enhance effects in co-operation with ICIs.

## 4. Modification of the Fibre Protein

Although most modifications to the adenovirus genome for oncolytic purposes have been within non-structural proteins, there have also been some efforts to alter the tropism of the virus by modifying capsid proteins and making infection more selective to tumor cells. The fiber protein has a critical role for the entry of adenoviruses in cells. With Ad5-based viruses the knob domain of the fiber protein first establishes a high affinity interaction with the cellular Coxsackie Adenovirus Receptor (CAR) [57], followed by an internalization induced via the binding of the RGD motif in the penton base to the alphaV integrins [58]. While the CAR is widely expressed in most cell types, it is poorly expressed in cells of hematopoietic origins [59] and is frequently lost in tumors [60,61,62,63,64,65]. The group B Adenoviruses, of which Ad3, 11 and 35 are members, utilize the CD46 and DSG-2 as receptors [66,67,68,69]. Both CD46 and DSG-2 [70] have been shown to be overexpressed in several tumors [71,72,73,74,75,76,77,78,79]. Thus, a strategy often used to ensure efficient infection of the oncolytic viruses in the tumor cells has been to modify the fiber gene. One approach has been to use a group B virus as backbone to generate the oncolytic viruses. Three viruses currently in clinical trials are based on a chimera of Ad11 and Ad3 with the fiber gene contributed by the Ad11 virus (see Table 1). Another approach has been to modify the fiber gene of the Ad5 serotype to contain the fiber shaft and knob domain of Ad35 [80], or the knob domain of Ad3 [22]. Another frequently used approach is to improve the entry of Ad5-based oncolytic viruses by cloning the RGD motif normally present in the Penton protein to the HI-loop of the fiber protein [81,82]. This modification reduces dependence on the CAR receptor by allowing the virus to utilize the RGD-integrin as an alternative pathway. Adding a polylysine (pK7) peptide to the C terminus of the RGD-fiber has been shown to further improve CAR-independent viral entry through the ability of the pK7 to bind polyanion motifs such as heparan sulfate, which are ubiquitously expressed on the surface of most cell types [83].

## 5. Modifications of the E3 Region

The E3 region of adenovirus is the least studied region of the virus, mostly because it was quickly found to be dispensable for virus replication in cultured cells [84]. Indeed, many early studies of adenoviruses were performed using the dl309 as a wild-type virus in which a major part of the E3 region was deleted as it was easier to make viral mutations in this background [85]. Most of the oncolytic viruses currently in clinical trials are of the Ad5 background, and the viruses of the Ad3/11 hybrid background has a deletion removing almost the entirety of the E3 region. As such, this section will focus on the E3 region of the Ad5 (group C) serotype. The E3 region of Ad5 expresses seven proteins that are thought to be immunomodulatory in function (reviewed in [86]). These properties make the E3 region of particular interest in the study of oncolytic viruses, considering that involvement of an immune response is beneficial for the success of oncolytic therapy. However, most of the oncolytic viruses currently in use have complete or partial deletions of the E3 region to facilitate the inclusion of transgenes. It remains to be determined if these E3 mutations are beneficial or detrimental to oncolytic or immunotherapy.

The proteins encoded on the E3 region have a wide range of effects on immune functions that can potentially enhance or inhibit oncoloytic activity (for an in-depth review, see [86]). The proteins encoded on E3 include: 12.5K, RIDa, RIDb, 6.7K, 14.7K, GP19K, and ADP. Remarkably, despite decades of research on Ad5, the functions of the E3-12.5K remain completely unknown and therefore its impact on oncolytic therapy cannot even be speculated without further investigation. The Receptor Internalization and Degradation proteins (RIDα and RIDβ) function as a heterodimer to block apoptosis induced by a variety of death ligands [87,88,89,90,91,92]. The RID complex also blocks TNF-mediated death [93,94] and may block EGFR-mediated inflammatory responses [95]. RID has also been shown to inhibit the interleukin 1- and TNF-induced NF-kB activation. The E3-6.7K is known as a general inhibitor of apoptosis, blocking both intrinsic and extrinsic pathways [96]. The E3-14.7K can protect cells from extrinsic apoptosis induced by some cytokines [86] and is a general inhibitor of TNF-mediated apoptosis [97,98]. E3-14.7K may also affect TNF-mediated inflammation as it inhibits TNF-induced activation of cPLA2 and thus the release of pro-inflammatory molecules [94,99]. The GP19K protein was shown to reduce the CTL-mediated killing of infected cells by blocking the transport of MHC class I molecules to the cell surface [100,101,102,103,104,105]. It does this, in part, by binding to and sequestering MHC class 1 molecules to the ER [101,102,104,106]. The final product of this region, the adenovirus death protein (ADP), is regulated by both the E3 promoter and the major late promoter and therefore reaches maximal expression in the late phase of infection. ADP kills infected cells by an unknown mechanism involving membrane degradation that is not apoptosis [107]. It is thought that this function of ADP helps with cell lysis and viral spread at the end of the infection cycle.

From the known functions of these E3 proteins it is possible to speculate which would be beneficial to leave in the oncolytic viruses and which could be deleted to make space for transgenes. To enhance viral spread and killing activity it would seem that expression of the ADP through the major late promoter should be maintained in oncolytic Ad vectors. Mutants of Ad that lack expression of ADP do not easily exit the cell, and methods such as freeze/thaw cycles need to be used to promote cell lysis and viral egress [84]. However, a recent study has shown that in some specific cases it may be better to remove ADP to achieve the best expression of transgene [108]. In this study the authors use viral vectors in which all E3 genes are removed except ADP. This results in increase expression of ADP and increased lysis of infected cells [109]. In addition, as their transgene (sodium iodide symporter, NIS) is a transmembrane protein they found that removal of the ADP gene resulted in better expression of NIS, and thus better imaging of infected tumor cells [108].

Conversely, since the RID complex and E3-14-7K appears to block inflammation it is likely that deleting these genes would be beneficial to increase immune killing of tumor cells. Similarly, since GP19K downregulates MHC complexes, deleting this gene would be essential to maintain immune cell killing of infected cancer cells. It is unfortunate that E3 remains one of the less studied transcription units of Ad since it will likely play a critical role in viral and immune system-mediated killing by oncolytic adenoviral vectors. Understanding the function of the E3-12.5K and its impact on tumor cell killing should also be prioritized since virtually nothing is currently known about this protein.

## 6. VA-RNAs

Adenoviruses produce two small, non-coding RNAs transcribed by RNA polymerase III, called VA-RNA I and VA-RNA II, that are approximately 160 nucleotide long [110]. They form a complex RNA structure that is stable and conserved, even if the actual sequence is not [111,112]. The structure is critical for binding and inhibiting an antiviral response protein, PKR [42,43,44]. These RNAs have been shown to be required for efficient viral replication [44,113,114,115] and the mechanism is at least in part due to its inhibition of PKR [43]. More recently, VA-RNAs were shown to activate the promoter of type 1 IFNs [116,117,118] sufficiently to induce expression of some IFN-induced genes; however, this does not happen by the classical pathway involving IRF-3 [117]. Although none of the currently used OAd vectors were designed with the status of VA-RNAs in mind, it may be a strategy to consider in the future. Whether to retain or remove the VA-RNAs is complicated by the still controversial role of PRK in cancer. Traditionally PRK has been considered a tumor suppressor since the mRNA is often downregulated in several cancer types (reviewed in [119]). In these tumors, deletion of the VA-RNAs may act as another factor to increase tumor selectivity. However, other studies have suggested an opposite effect of PRK and it has been shown to be overexpressed in some tumors [120,121,122,123]. The case for deletion of VA-RNAs in oncolytic Ad vectors may therefore depend upon the cancer type being targeted and the expression of PKR in these cell types.

## 7. Conclusions

The application of adenovirus as an oncolytic viral therapy is entering an exciting phase. The combination of these vectors with ICIs is generating provocative results in the clinic and may provide a solution to improve response rates to immunotherapy. However, most of the existing Ad vectors have not been optimized to remove viral genes that attenuate immune and innate anti-viral responses. With the wide range of ongoing clinical trials using Ad vectors, a clearer view may begin to emerge regarding which alterations of viral genes are best to enhance oncolytic properties of the virus and to enhance anti-tumor immune responses. In particular, further research into the E3 transcription unit and its role in suppressing immune cell killing may provide important insights in understanding the interactions of Ad-infected cells with the immune system, and how these effects influence oncolytic virotherapy and immunotherapy.

## Figures and Tables

**Figure 1:**
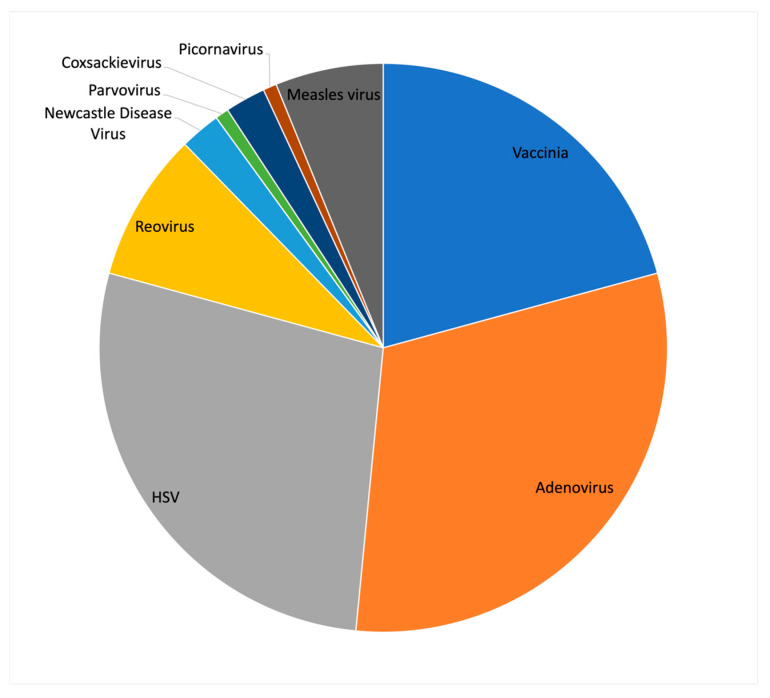
Summary of virus types currently in clinical trials as oncolytic agents. (Data source: clinicaltrials.gov, accessed on 1 October 2022).

**Table 1:** Adenoviral vectors currently in clinical trials for cancer therapy. Replication defective viruses that are used for gene delivery only are shaded in grey.

Name	Serotype Based on	E1A	Fiber	Transgene	E3 Status	Other Modification	References
H101 (oncorin)	Ad5	untouched	Ad5	none	del of 78.3-85.8 m.u. Probably only E3-12.5K left	deletion of E1B55K	[8]
ADV/HSV-tk	Ad5 viral vector	transgene in deleted E1	Ad5	HSV tk gene with the RSV LTR promoter	Deletion of RIDα, RIDβ and E3-14.7K		[9]
MEM-288	Ad5	delta 24	Ad5	chimeric CD40 ligand and IFNb	deleted	deletion of E1B55K	[10]
LOAd703	Ad5	E2F promoter, delta 24	chimeric Ad5/Ad35	TMZ-CD40L + 4-1BBL	deleted E3-6.7K and gp19K		[11,12,13]
CG0070	Ad5	E2F promoter	Ad5	GM-CSF under control of E3 promoter	gp19K deleted		[14]
AdAPT-001	Ad5	50bp del in E1A promoter (tumor specific)	Ad5	mTGFbR-IgG (TGFb trap)	Deletion of RIDα, RIDβ and E3-14.7K	deletion E1B19K	[15,16]
OBP-301 (Telomelysin)	Ad5	human TERT promoter E1A-IRES-E1B in E1 deleted backbone	Ad5	none	deleted		[17,18]
Colo-Ad1 (Enadenotucirev)	Ad11/Ad3 chimera	untouched (Ad11)	Ad11	none	near complete deletion	deletion in E4orf4 gene	[19]
DNX-2440	Ad5	delta 24	Ad5 with RGD-motif into the H-loop	OX40 ligand	deleted		[20,21]
CAdVEC	Ad5 + Ad5 gutted vector	delta24	Ad5	undisclosed immunomodulatory molecules in vector	deleted		
CGTG-102(ONCOS-102)	Ad5	delta 24	Ad5 with fiber knob of Ad3	GM-CSF under control of E3 promoter	E3-6.7K and gp19K deleted		[22,23,24]
DNX-2401	Ad5	delta 24	Ad5 with RGD-motif into the H-loop	none	deleted		[21]
TILT-123	Ad5	E2F promoter, delta 24	Ad5 with fiber knob of Ad3	TNFa and IL-2	transgenes in E3 unclear what is deleted		[25]
Ad-E6E7	Ad5 viral vector (vaccine)	E1 deleted	Ad5	attenuated fusion E6E7 transgene	deleted	+ Maraba virus	[26]
Ad-MAGEA3	Ad5 viral vector (vaccine)	E1 deleted	Ad5	MAGE-A3	deleted	+ Maraba virus	[27]
VCN-01	Ad5	E2F promoter, delta 24	Ad5 with RGDK motif in shaft	soluble version of human PH20 under late promoter	WT		[28]
NG-350A	Ad11/Ad3 chimera	untouched (Ad11)	Ad11	heavy and light chains for a secreted CD40 agonist monoclonal antibody	near complete deletion		PsiOxus Therapeutics
NG-641	Ad11/Ad3 chimera	untouched (Ad11)	Ad11	secreted Interferon alpha, the chemokines CXCL9, CXCL10 and an anti-FAP/anti-CD3 bispecific T-cell activator	near complete deletion		PsiOxus Therapeutics
cells with CRAd-survivin-pk7 virus	Ad5	human survivin promoter	Ad5 modified with polylysine	none	WT		[29]
ORCA-010	Ad5	delta 24	Ad5 with RGD-motif into the H-loop	none	mutated GP19K	T1 mutation in E3-19K	[30]

**Table 2:** List of clinical trials currently using oncolytic Adenoviral vectors as oncolytic agents. (Data source: clinicaltrials.gov, accessed on October 2022).

Trial Identifier	Phase	Conditions	Virus Name	E3 Status	Additional Treatment
NCT05051696	N/A	Genital Neoplasms, Female	Oncorine (H101)	only 12.5K present	+/- radiotherapy
NCT04771676	2	Refractory Malignant Ascites	Oncorine (H101)	only 12.5K present	none
NCT03004183	2	Metastatic Non-small Cell Lung Cancer, Metastatic Triple-negative Breast Cancer	ADV/HSV-tk	deletion of RIDα/β and 14.7K	Valacyclovir, SBRT, Pembrolizumab
NCT05076760	1	Advanced Solid Tumors	MEM-288	deleted	MEM-288 Intratumoral Injection
NCT02705196	1/2	Pancreatic Cancer	LOAd703	deletion of 6.7K and gp19K	gemcitabine, nab-paclitaxel, atezolizumab
NCT02143804	2	High Grade, Bladder Cancer, Non Muscle Invasive	CG0070	gp19K deletion	none
NCT02365818	2	Bladder Cancer	CG0070	gp19K deletion	CG0070
NCT05234905	2	Uterine Cervical Neoplasms	Oncorine (H101)	only 12.5K present	Camrelizumab
NCT05113290	4	Hepatocellular Carcinoma	Oncorine (H101)	only 12.5K present	Sorafenib
NCT05124002	4	Cholangiocarcinoma, Intrahepatic	Oncorine (H101)	only 12.5K present	HAIC or FOLFOX
NCT04673942	1	Refractory Solid Tumor, Adult	AdAPT-001	deletion of RIDα/β and 14.7K	none
NCT03190824	2	Melanoma Stage III and iv	OBP-301 (Telomelysin)	deleted	none
NCT01438112	2	Non Muscle Invasive Bladder Cancer	CG0070	gp19K deletion	none
NCT03916510	1	Locally Advanced Rectal Cancer	Enadenotucirev (previously ColoAd-1)	near complete deletion	Capecitabine, Radiotherapy
NCT03714334	1	Glioblastoma	DNX-2440	deleted	none
NCT03740256	1	Various solid tumors	CAdVEC	deleted	none
NCT01437280	1	Solid Tumors	CGTG-102	6.7K and gp19K deleted	none
NCT05180851	1	Various solid tumors	Recombinant L-IFN adenovirus	unknown	none
NCT02197169	1	Glioblastoma or Gliosarcoma	DNX-2401	deleted	Interferon-gamma
NCT02053220	1	Solid Cancers	Colo-Ad1	near complete deletion	none
NCT02028442	1/2	Solid Tumors of Epithelial Origin	Enadenotucirev (previously ColoAd-1)	near complete deletion	none
NCT02028117	1	Recurrent Platinum Resistant Ovarian Cancer	Enadenotucirev (previously ColoAd-1)	near complete deletion	none
NCT05222932	1	Melanoma, Head and Neck Squamous Cell Carcinoma	TILT-123	unknown deletion	Avelumab
NCT01598129	1	Malignant Solid Tumor	CGTG-102	6.7K and gp19K deleted	low-dose metronomic cyclophosphamide.
NCT02798406	2	Brain Cancers	DNX-2401	deleted	pembrolizumab
NCT04217473	1	Metastatic Melanoma	TILT-123	unknown deletion	TILT-123
NCT03618953	1	HPV-Associated Cancers	Ad-E6E7, MG1-E6E7	deleted	Atezolizumab
NCT04695327	1	Solid Tumor	TILT-123	unknown deletion	TILT-123
NCT03773744	1	Metastatic Melanoma, Squamous Cell Skin Carcinoma	Ad-MAGEA3, MG1-MAGEA3	deleted	Pembrolizumab, Cyclophosphamide
NCT02045602	1	Locally Advanced Solid Tumors	VCN-01	WT	Gemcitabine, Abraxane^®^
NCT02045589	1	Pancreatic Adenocarcinoma	VCN-01	WT	Gemcitabine, Abraxane^®^
NCT02879760	1/2	Non-Small Cell Lung Cancer	Ad-MAGEA3, MG1-MAGEA3	deleted	Pembrolizumab
NCT04685499	2	Head and Neck Squamous Cell Carcinoma With Inoperable Recurrent or Progressive Disease	OBP-301	deleted	Pembrolizumab
NCT03225989	1/2	Solid Cancers	LOAd703	deletion of 6.7K and gp19K	none
NCT03896568	1	High Grade Glioma	DNX-2401	deleted	none
NCT01956734	1	Glioblastoma Multiforme	DNX-2401	deleted	Temozolomide
NCT03178032	1	Brainstem Glioma	DNX-2401	deleted	none
NCT03852511	1	Metastatic Cancer	NG-350A	near complete deletion	none
NCT04053283	1	Metastatic Cancer	NG-641	near complete deletion	none
NCT03072134	1	Malignant Glioma	Neural stem cells loaded with NSC-CRAd-Survivin-pk7	WT	none
NCT04097002	1/2	Adenocarcinoma of the Prostate	ORCA-010	mutated gp19K	none
NCT05561491	2	Melanoma	ONCOS-102 (Previously known as CGTG-102)	6.7K and gp19K deleted	Balstilimab

## Data Availability

Not applicable.
